# Low-Dose Vericiguat in Heart Failure With Reduced Ejection Fraction: A Case Report

**DOI:** 10.7759/cureus.99626

**Published:** 2025-12-19

**Authors:** Chinedu Orji

**Affiliations:** 1 Cardiology, North Cumbria Integrated Care, Whitehaven, GBR

**Keywords:** cardiovascular side effects, chronic kidney disease (ckd), guideline-directed medical therapy (gdmt), heart failure pharmacological treatment and devices, heart failure with reduced ejection fraction

## Abstract

Vericiguat is used as an adjunct therapy for patients with heart failure with reduced ejection fraction (HFrEF). Despite its availability in clinical practice, it remains underutilized among eligible patients. This case report describes the clinical benefit of low-dose vericiguat as an add-on therapy in a patient with worsening symptoms due to decompensated HFrEF.

A 78-year-old man with HFrEF secondary to ischemic cardiomyopathy experienced recurrent decompensation despite maximally tolerated guideline-directed medical therapy. Vericiguat was initiated at 2.5 mg daily and was not up-titrated due to concerns regarding borderline low blood pressure and worsening renal function. Following treatment initiation, the patient demonstrated symptomatic improvement, with a New York Heart Association (NYHA) functional class improvement from III to I, an increase in left ventricular ejection fraction, and a reduction in heart failure-related hospital admissions during follow-up. Renal function remained stable, and no adverse effects, including hypotension or electrolyte abnormalities, were observed.

Despite advances in guideline-directed therapy, outcomes remain suboptimal for many patients with HFrEF. This case highlights the potential clinical benefit of low-dose vericiguat as a safe and effective adjunct therapy, with improvement in functional status and reduced hospitalization burden without compromising renal function. However, these observations may also reflect other contributing factors, and causality cannot be established. Further studies are needed to better define the long-term safety and efficacy of lower-dose vericiguat in broader patient populations.

## Introduction

Chronic heart failure (CHF) is a progressive clinical syndrome associated with high morbidity, mortality, and healthcare utilization worldwide. Heart failure with reduced ejection fraction (HFrEF) defined as heart failure with a left ventricular ejection fraction (LVEF) ≤40% carries a particularly poor prognosis, with frequent hospitalizations despite advances in guideline-directed medical therapy (GDMT) [1].

Contemporary management of HFrEF relies on a combination of disease-modifying therapies, including angiotensin receptor-neprilysin inhibitors (ARNIs), beta-blockers (BBs), mineralocorticoid receptor antagonists (MRAs), and sodium-glucose cotransporter 2 (SGLT2) inhibitors. Although these therapies improve survival and reduce hospitalizations, many patients are unable to tolerate full-dose GDMT due to hypotension, renal dysfunction, advanced age, or comorbidities, and only a minority achieve target doses in real-world practice. In a recent cohort study [2], only 3.6% of patients receiving therapy were on SGLT2 inhibitors, and only a small proportion achieved target doses of multiple therapies simultaneously.

Vericiguat is a novel oral soluble guanylate cyclase (sGC) stimulator that targets the impaired nitric oxide (NO)-sGC-cyclic guanosine monophosphate (cGMP) signaling pathway in heart failure. By directly stimulating sGC and increasing cGMP production, vericiguat improves vascular function and counteracts maladaptive cardiac remodeling [3]. Clinical evidence from the SOCRATES-REDUCED and VICTORIA trials showed reduced cardiovascular death and heart failure hospitalization in a high-risk population with recently decompensated HFrEF [4].

Despite approval by the US Food and Drug Administration (FDA) in 2021 [5], real-world use of vericiguat among eligible patients remains limited [6]. Nearly half of the untreated patients in one study [7] were eligible for therapy; however, most did not reach the target dose [8] or were initiated only at advanced stages of disease. This suggests limited clinician familiarity and highlights the challenges of optimizing medical therapy in this population. This case report aims to illustrate the clinical benefit and tolerability of low-dose vericiguat in a high-risk elderly patient with HFrEF, chronic kidney disease (CKD), and borderline blood pressure, highlighting its potential role when full-dose GDMT is not feasible or effective.

## Case presentation

A 78-year-old man was evaluated in October 2021 for atrial flutter after reporting chest discomfort the previous week. He had no cognitive impairment but was noted to be frail, with reduced functional capacity for strenuous tasks. His medical history included hypertension, hyperlipidemia, gastroesophageal reflux disease, and benign prostatic hyperplasia. Home medications included tamsulosin, triamterene/hydrochlorothiazide (37.5/25 mg), donepezil (10 mg), amlodipine (10 mg), atorvastatin (20 mg), and pantoprazole (40 mg).

**Figure 1:**
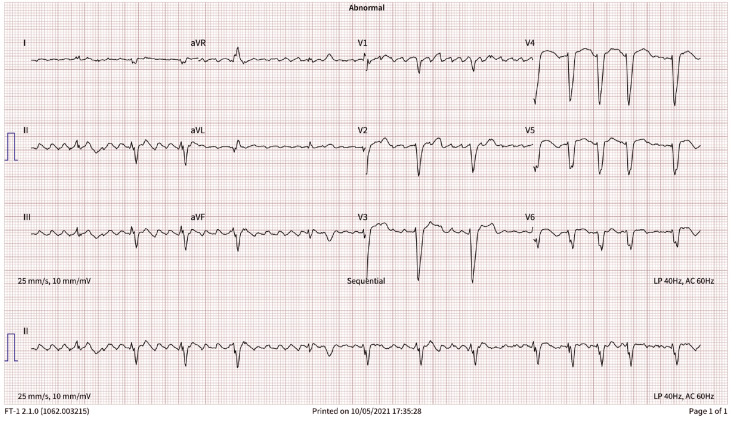
ECG in October 2021 ECG: electrocardiography; aVR: augmented vector right; aVL: augmented vector left; aVF: augmented vector foot

Initial assessment revealed atrial flutter with a ventricular rate of 85 beats per minute and frequent premature ventricular complexes (PVCs) on electrocardiography (ECG) (Figure 1, ECG in October 2021). Transthoracic echocardiography (TTE) demonstrated an LVEF of 50-55% with abnormal diastolic function. Serum creatinine was 1.63 mg/dL, corresponding to an estimated glomerular filtration rate (eGFR) of 53 mL/min/1.73 m². He was started on apixaban 5 mg twice daily and metoprolol 25 mg twice daily. After a detailed discussion with the patient, he elected the medical management of his rhythm abnormality. Further evaluation was planned; however, he traveled abroad and was subsequently lost to follow-up.

**Figure 2:**
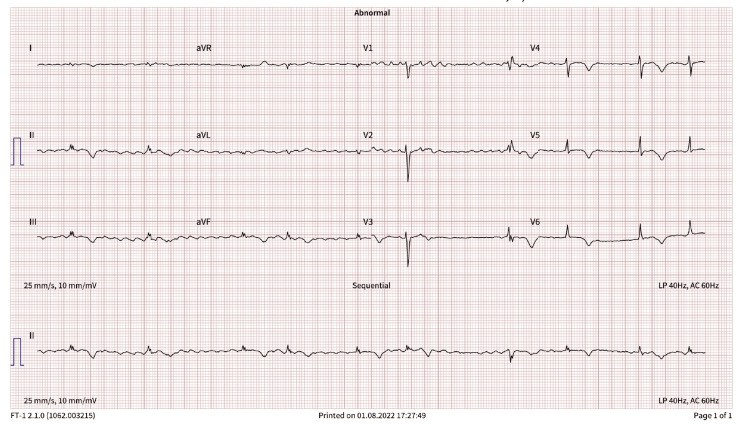
ECG in March 2022 ECG: electrocardiography; aVR: augmented vector right; aVL: augmented vector left; aVF: augmented vector foot

He re-presented in March 2022 after multiple admissions for decompensated heart failure. His medications were revised in his home country to sacubitril/valsartan (49/51 mg twice daily), furosemide (20 mg daily), rivaroxaban (20 mg daily), and spironolactone (25 mg daily); apixaban and triamterene/hydrochlorothiazide were discontinued. Subsequent ECG showed atrial fibrillation with T-wave abnormalities, a heart rate of 55, and no PVCs (Figure 2, ECG in March 2022). Repeat TTE revealed severely reduced left ventricular systolic function with global hypokinesis and an ejection fraction of 20-25%, and serum creatinine was 1.8 mg/dL (eGFR 38 mL/min/1.73 m²). He was diagnosed with chronic systolic HFrEF, classified as New York Heart Association (NYHA) Class II, atrial fibrillation, and coronary artery disease (CAD). Dapagliflozin (10 mg daily) was added. Coronary angiography showed mild to moderate CAD, managed medically.

By July 2023, follow-up TTE showed improvement in ejection fraction to 30% with symptom improvement to NYHA Class I. However, renal function subsequently declined (serum creatinine 2.02 mg/dL, eGFR 33 mL/min/1.73 m²), accompanied by hyperkalemia. Spironolactone was discontinued, and rivaroxaban was replaced with apixaban. Over the following months, his condition worsened to NYHA Class III. He reported shortness of breath during ordinary activities, with significant lower extremity swelling. Intensified diuretic therapy and reintroduction of spironolactone did not prevent recurrent hospitalizations. By February 2024, TTE demonstrated further deterioration in systolic function with an ejection fraction of 15-20% (Table 1, clinical parameters over time).

**Table 1:** Clinical parameters over time HFrEF: heart failure with reduced ejection fraction; LVEF: left ventricular ejection fraction; NYHA: New York Heart Association; SBP: systolic blood pressure; eGFR: estimated glomerular filtration rate

Timeline	LVEF (%)	NYHA Class	SBP (mmHg)	Creatinine (mg/dL)	eGFR (mL/min/1.73 m²)
Oct 2021 (first presentation)	50-55	-	124	1.63	53
Mar 2022 (HFrEF diagnosis)	20-25	II	103	1.8	38
July 2023 (partial recovery)	30	I	98	2.02	33
Feb 2024 (deterioration)	15-20	III	96	2.0	34
May 2024 (vericiguat start)	-	III	105	-	-
Aug 2024 (follow-up)	30	I	102	2.02	33

**Figure 3:**
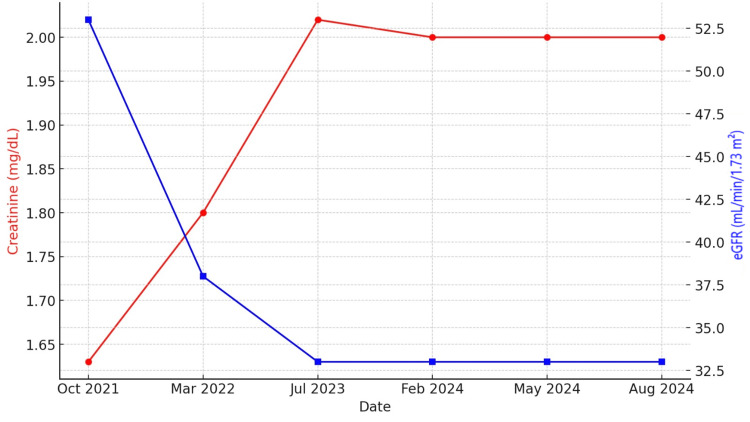
Creatinine and eGFR trend eGFR: estimated glomerular filtration rate

In May 2024, due to repeated episodes of CHF exacerbation, vericiguat was initiated at 2.5 mg once daily. At the time of initiation, he had a low-normal systolic blood pressure (SBP) of 105 mmHg and a normal heart rate. Renal function and blood pressure were monitored every 2-4 weeks during follow-up. After three months of therapy, he demonstrated marked clinical improvement, with resolution of dyspnea and only mild leg swelling noted. He had no further hospitalizations during this period, and repeat TTE showed an increase in LVEF to 30%. Renal function (Figure 3, creatinine and eGFR trends over time) and blood pressure remained stable, and no adverse effects were reported. Given his clinical improvement, advanced age, prior renal impairment, and low-normal blood pressure, a risk-benefit assessment supported maintaining vericiguat at the low dose.

## Discussion

In healthy individuals, endothelial-derived NO binds to sGC, activating the conversion of guanosine triphosphate (GTP) to cGMP. This signaling cascade promotes vascular smooth muscle relaxation, decreases cardiac workload, and limits ventricular hypertrophy, inflammation, and fibrosis. Impairment of the NO-sGC-cGMP pathway is a recognized feature of heart failure [3]. Vericiguat, an oral stimulator of sGC, is currently the only available therapy that directly targets this pathway, acting both independently of NO and synergistically to maintain cGMP availability [9].

Several clinical trials, including SOCRATES-REDUCED, SOCRATES-PRESERVED, VITALITY, and VICTORIA [4], have demonstrated that vericiguat improves patient-reported health status, reduces hospitalizations, and lowers all-cause mortality in individuals with ejection fraction below 45%, with minimal renal adverse effects. Clinical guidelines subsequently recommended vericiguat as adjunct therapy for patients with symptomatic HFrEF below 45% who remain at high risk despite standard management [10].

The clinical course observed in this patient aligns with these outcomes. Following vericiguat initiation, he experienced improvement in NYHA functional class from III to I, absence of further hospitalizations, stable renal function, and no major adverse effects. Additionally, the improvement in ejection fraction supports a possible reverse remodeling effect, consistent with findings from prior studies [11] showing clinically meaningful increases in ejection fraction with vericiguat therapy, irrespective of background GDMT. However, these observations should be interpreted with caution. Improvement may also reflect confounding factors, including the optimization of background therapy, heart rate or rhythm control, and delayed effects of prior medications. Causality cannot be definitively attributed to vericiguat alone.

This case specifically underscores the potential role of low-dose vericiguat in real-world practice. In pivotal trials, vericiguat was initiated at 2.5 mg once daily and up-titrated every two weeks to a target dose of 10 mg, contingent on blood pressure and renal stability [7]. In contrast, dose escalation was intentionally avoided in this patient due to advanced age, CKD, low-normal systolic blood pressure, and a prior history of hyperkalemia with MRA therapy. These factors mirror real-world observations, where older adults and patients with renal dysfunction frequently remain on lower-than-target doses or are unable to complete titration. Observational data consistently show that only a minority of eligible patients achieve target dosing of newer heart failure therapies [8], highlighting a gap between trial protocols and clinical practice. This case suggests that meaningful clinical benefit may still be achieved with sustained low-dose vericiguat when full titration is not feasible, supporting its pragmatic use in high-risk, elderly populations.

## Conclusions

Vericiguat, although relatively new in heart failure management, shows potential as an adjunct therapy for patients with decompensated HFrEF. In this patient, low-dose vericiguat was temporally associated with improvement in cardiac function, ejection fraction, and symptoms over the short term. Given the presence of potential confounders and the limitations of single-patient observation, causality cannot be established. Further research, including randomized clinical trials and prospective cohort studies, is warranted to evaluate the efficacy, safety, and optimal dosing of vericiguat in broader, high-risk patient populations.
